# Supplementary material for: Complexome profiling of the Chlamydomonas psb28 mutant reveals TEF5 as an early PSII assembly factor
Source: Plant Cell. 2025 Mar 17;37(6):koaf055. doi: 10.1093/plcell/koaf055 (PMC12164590; doi:10.1093/plcell/koaf055)
Supplement: koaf055_Supplementary_Data [file koaf055_supplementary_data.zip › psb28_interactive.zip!/plots/intensity/qualityPlots/peptides/FTSH2_Cre17.g720050.t1.2.html]

### Cre17.g720050.t1.2

Trivial name: FTSH2  
SeqCoverage [median]: 39.80  
Mass: 74.38 kDa  
  
 

| Sequence | MissedCleavage | Mass [Da] | wtIntensity | muIntensity | Score |
| --- | --- | --- | --- | --- | --- |
| AAEELVFGEDEVTSGAASDLQQVSGMAR | 0 | 2866.33 | 18144.85 | 63056.25 | 114.74 |
| ADILDPALLRPGR | 1 | 1405.80 | 42117.10 | 76189.73 | 83.31 |
| ADILDPALLRPGRFDR | 2 | 1824.00 | 20006.29 | 134339.33 | 73.02 |
| AITNKEIDDAIDR | 1 | 1472.75 | 44538.39 | 92933.28 | 134.68 |
| AMLAEYTTIPEENVK | 0 | 1707.84 | 61236.22 | 187297.65 | 199.68 |
| AMLAEYTTIPEENVKAVEAQR | 1 | 2362.18 | 574.28 | 4674.89 | 85.64 |
| EIDDAIDR | 0 | 945.44 | 28631.29 | 49360.02 | 121.31 |
| ETLTGDEFR | 0 | 1066.49 | 1822.09 | 3689.96 | 80.22 |
| FLEYLEMGR | 0 | 1156.56 | 79705.52 | 264706.68 | 138.78 |
| FTAVGAR | 0 | 720.39 | 58738.18 | 117524.12 | 135.50 |
| GLTWFIPGEDPTLVSK | 0 | 1758.92 | 3268.75 | 0.00 | 65.50 |
| GTGIGGTNDER | 0 | 1075.49 | 19049.32 | 23020.41 | 142.98 |
| HIADNREAIDR | 1 | 1308.65 | 9565.86 | 26687.52 | 69.98 |
| IDSQVR | 0 | 716.38 | 39164.03 | 122869.48 | 170.31 |
| IVAGLEGKPLVDGK | 1 | 1394.81 | 116597.98 | 194500.29 | 130.47 |
| IVEALMEK | 0 | 931.50 | 1177.49 | 6092.03 | 91.32 |
| IVEALMEKETLTGDEFR | 1 | 1979.99 | 47777.51 | 227016.29 | 216.93 |
| IVGALGGR | 0 | 741.45 | 127814.73 | 312980.46 | 150.06 |
| KVAEDVDLQEVAMR | 1 | 1601.81 | 554.63 | 8052.80 | 116.84 |
| NSMSESLQQR | 0 | 1178.54 | 23089.74 | 34725.22 | 198.61 |
| QVSVDLPDQK | 0 | 1127.58 | 81773.29 | 84728.01 | 259.86 |
| QVSVDLPDQKGR | 1 | 1340.70 | 626.79 | 3837.16 | 52.58 |
| SQIFAR | 0 | 720.39 | 19281.72 | 50007.62 | 119.22 |
| TIADQAYEVALR | 0 | 1348.70 | 167019.88 | 491663.75 | 225.22 |
| TPGFAGANLMNLLNEAAILAGR | 0 | 2213.16 | 1011.72 | 0.00 | 122.77 |
| VAEDVDLQEVAMR | 0 | 1473.71 | 87158.49 | 140008.77 | 257.01 |
| VQLPGTSPELLGK | 0 | 1337.76 | 133629.52 | 315436.90 | 140.78 |
| VQLPGTSPELLGKFR | 1 | 1640.92 | 0.00 | 685.63 | 31.83 |
| VRVQLPGTSPELLGK | 1 | 1592.92 | 30389.08 | 149094.32 | 131.53 |
|  |  |  | 1264464.7 | 3185178.5 |  |
